# Protocol for north of England and Scotland study of tonsillectomy and adeno-tonsillectomy in children (NESSTAC). A pragmatic randomised controlled trial comparing surgical intervention with conventional medical treatment in children with recurrent sore throats

**DOI:** 10.1186/1472-6815-6-13

**Published:** 2006-08-09

**Authors:** John Bond, Janet Wilson, Martin Eccles, Alessandra Vanoli, Nick Steen, Ray Clarke, Andrew Zarod, Catherine Lock, Katie Brittain, Chris Speed, Nikki Rousseau

**Affiliations:** 1 Centre for Health Services Research, Newcastle University, Newcastle upon Tyne, UK; 2 ENT, Freeman Hospital, Newcastle upon Tyne, UK; 3 ENT, Alder Hey Children's Hospital, Liverpool, UK; 4 ENT, Booth Hall Children's Hospital, Manchester, UK

## Abstract

**Background:**

Uncertainties surrounding the effectiveness and cost-effectiveness of childhood tonsillectomy for recurrent sore throat led the NHS Health Technology Assessment Programme to commission this research to evaluate the effectiveness and cost-effectiveness of tonsillectomy and adeno-tonsillectomy in comparison with standard non-surgical management in children aged under 16 with recurrent throat infections. The aim is to evaluate if tonsillectomy and adeno-tonsillectomy reduces the number of episodes of sore throats among children to a clinically significant extent.

**Methods/design:**

A simple prospective pragmatic randomised controlled trial with economic analysis and prospective cohort study of non-trial participants comparing surgical intervention with conventional medical treatment. The treatment arm will receive tonsillectomy and adeno-tonsillectomy while in the control arm non-surgical conventional medical treatment only will be used. The primary outcome measure will be reported number of episodes of sore throat over two years with secondary outcomes measures of reported number of episodes of sore throat, otitis media and upper respiratory tract infection which invoke a GP consultation; reported number of symptom-free days; reported severity of sore throats and surgical and anaesthetic morbidity. The study will take place in five hospitals in the UK. The trial population will be 406 children aged 4–15 on their last birthday with recurrent sore throat referred by primary care to the 5 otolaryngology departments. The duration of the study is seven years (July 2001- July 2008).

**Discussion:**

As with all pragmatic randomised controlled trials it is impossible to control the external environment in which the research is taking place. Since this trial began a number of factors have arisen which could affect the outcome including; a reduction in the incidence of respiratory tract infections, marked socio-economic differences in consultation rates, the results from the National Prospective Tonsillectomy Audit and the Government's waiting list initiatives.

## Background

In the UK sore throats cost the NHS an estimated £60 million in GP consultations alone, result in 90,000 tonsillectomy procedures, approximately half of which are in children, and a loss of more than 35 million school or work days annually[1]. The incidence of tonsillectomy has risen since the early 1990's, although levels are still much lower than in the 1930's, when 100,000 operations were performed in UK school children[2]. Adenoidectomy is performed with tonsillectomy in about one third of patients. Private medical insurance is associated with higher selective ENT surgical rates under the age of seven years[3] and 16% of UK ENT activity is in the independent sector. Therefore figures based purely on NHS returns inevitably underestimate the total activity. In addition to the health care costs, tonsillectomy incurs parental costs as one parent usually resides in hospital overnight. Thereafter the average time to return to normal activity for under 15 year olds is 12 days[4].

There is a broad similarity in the criteria for tonsillectomy in clinical guidelines in the UK [5,6] and North America[7]. The minimum criteria are typically a two year history of three to four sore throats of moderate severity (five day duration) per annum. This is despite evidence that even histories that seem impressive may not be confirmed on close scrutiny in the majority[8]. The complex psychosocial influences on tonsillectomy rates include parental enthusiasm for intervention[9], lack of information[10] and maternal use of psychotropic drugs which increases two-fold the rate of consultation for childhood sore throat [11,12]. Guidelines may not be uniformly implemented, even when locally derived. Surgeons tend to break guidelines more often in favour of performing than withholding surgery[5].

National and international variations in the rates of adeno-tonsillectomy have been recognised for decades. Even in the 1930's, 50% of UK and USA children received a tonsillectomy, while the rate was 0.5% or lower in Germany[2]. A survey of such variation in Quebec, highlighted the importance of clinical uncertainty among physicians about the recommendation of surgical intervention[13], providing further support for conducting primary research. The Scottish National Tonsil Audit showed that rates of tonsillectomy in childhood varied from <4/10,000 in Forth Valley to almost 10/10,000 in Dumfries and Galloway[14].

Differential costs and benefits of surgery at different age groups are not known. The tonsils are traditionally thought to undergo a period of physiological enlargement around school entry. At this time also, pathological sequelae may include otitis media. Older children and adolescents, may have a somewhat different natural history, and illness at this age has rather different (educational) implications.

Mortality from tonsillectomy has been estimated at 1/16000 to 1/35000[15], but surgical risk at this level is hard to measure, to conceptualise and to convey. The major nonfatal complications are infection, haemorrhage (2.15%), and pain which lasts on average five to six days [16,17] and may be inadequately treated in children[18]. Haemorrhage is unpleasant, requires intravenous fluid administration, with or without blood transfusion and return to theatre. The reported rate of second anaesthetic for haemostasis varies widely from 0.75% in one British review[4], to as low as 0.06% in a study of almost 9409 children in Toronto[19]. The post tonsillectomy readmission rate is up to 7%[4], but in Newcastle in childhood is only 2.3% (unpublished data; Department of Clinical Effectiveness, Freeman Hospital, Newcastle). The overall reported complication rate ranges from 8%[14] to 14%[17], the majority being relatively minor such as sore throat, nausea, fever, dysphagia. Most two to 10 year olds undergoing ENT surgery show behavioural changes such as attention seeking, temper tantrums and night waking and there is also anecdotal evidence for depression after tonsillectomy[20]. Younger children, due to cognitive immaturity seem less well able to adapt to hospitalisation [21,22]. Late sequelae may include lower postoperative serum immunoglobulin levels but these have been ascribed to reduction in antigen stimulation[23]. There is continuing debate about the suggestion that tonsillectomy increases the risk of Hodgkin's lymphoma[24]. A substantial Scandinavian population based cohort study found an increased risk of Hodgkin's disease, especially in younger children[25]. The risk of transmission of nvCJD from contaminated tonsillectomy instruments remains quite unquantified. Some centres are costing the use of disposable tonsillectomy sets.

Despite the frequency of tonsil dissection, there is a remarkable lack of robust evidence for its efficacy. Uncontrolled patient reports suggest the procedure to be very effective but recurrent sore throat, particularly in childhood may be a self limiting disease. Where non-intervention control groups have been studied, the benefits of tonsillectomy seem almost to disappear after two years. Available studies are either 20 to 30 years old or confined to small numbers of severely affected individuals with limited general applicability. The most recently published Cochrane review concludes that there is no evidence from randomised controlled trials to guide the clinicians in formulating the indications for surgery in children or adults[26]. The authors state the need for high quality evidence from randomised controlled trials to establish its effectiveness and that these should assess the effectiveness of the procedure in patients with throat infections of differing severity and frequency. A recent Dutch randomised controlled trial of adenotonsillectomy versus watchful waiting reported no differences between treatment arms for children with mild symptoms and only a small difference of less than one episode of fever a year between treatment arms for children with moderate symptoms[27].

The Scottish National Tonsillectomy Audit[14] showed high levels of patient satisfaction and that 80% of subjects did not consult a doctor in the subsequent 12 months. However, over the past 30 years a number of controlled studies with longer follow-up indicate marginal and diminishing levels of clinical benefit over a period of non-intervention. There are no substantial claims for the benefit of childhood tonsillectomy after 2 years. Roos[28] assessed the benefit to be 1 to 1.5 fewer sore throats (0.5 to 1 episode per annum) over the first two years after surgery in those with three to four episodes per annum preoperatively. Other studies [29-31] showed benefits of the order of ~1.5 fewer sore throats versus controls in the first postoperative year and on average one fewer episode in the second year. All of these and other available studies provide inadequate evidence because of poor definition of entry and outcome criteria, failure to include intention to treat calculations and small or skewed samples[32]. Even the only scientifically acceptable study by Paradise and colleagues[17] suffered from comparatively small numbers of a skewed population of more severely affected children. The benefits of surgery were more marked (approximately 1.75 fewer episodes in year 1, 1.5 in year 2) but equally short lived. The drop out rate was 34% by the end of year 2 and 1 in 3 of the control group underwent surgery and were excluded from analysis. Also, the very active therapy of the control arm may have mitigated any impact of surgery. The Paradise group went on to study a more typical i.e. less severely affected group of children, but the full results of this study, near completion in 1992 have never been reported.

Weight gain is a cited supplementary benefit of tonsillectomy. Two recent studies showed accelerated weight gain postoperatively, but as the children were shown to be of normal or above average height and weight preoperatively, this effect may be undesirable[33]. There appears so far to be only minimal additional benefit from adenoidectomy or adenotonsillectomy in recurrent acute otitis media [16].

A straw poll, for this protocol, of consultant otolaryngologists asked: what level of reduction in sore throat would justify removal of the tonsils? Replies were remarkably consistent – at least 2 sore throats fewer per annum. No published trial to date shows a benefit of this magnitude, even in the first year after surgery. There is a pressing need for a UK, pragmatic trial to evaluate the effectiveness and cost-effectiveness of childhood tonsillectomy.

The purpose of this study therefore is to answer the key research question "What is the effectiveness and cost-effectiveness of tonsillectomy/adeno-tonsillectomy in comparison with standard non-surgical management in children aged under 16 with recurrent throat infections?" Assessment of outcome will emphasise those which are important to children themselves and their parents or carers. Specific research questions are;

• Does tonsillectomy/adeno-tonsillectomy reduce the number of episodes of recurrent sore throats among children to a clinically significant extent?

• Are there differences in clinical outcome for the age groups: 4–7, 8–11, 12–15 years?

• What is the cost effectiveness of tonsillectomy/adeno-tonsillectomy among children and what are the costs and benefits to families?

• What are the important outcomes of tonsillectomy/adeno-tonsillectomy for children and their parents/carers and what is the importance of these to children and their parents' quality of life?

• What are parents' (and older children's) preferences for different treatment options for recurrent sore throat?

• How representative of the target population are trial participants?

## Methods/design

### Trial design

A simple prospective pragmatic randomised controlled trial with economic analysis comparing surgical intervention with conventional medical treatment.

### Cohort design

We anticipate that a large majority of participants who decline randomisation to the trial will opt for, and receive, surgery. Therefore, in order to assess the external validity of the trial results, we will recruit a cohort of children from those who decline to participate in the trial. The cohort will include both children who opt for surgery and those who choose conventional medical treatment. They will be followed up for 24 months.

### Interventions

The treatment arm will receive tonsillectomy and adeno-tonsillectomy while in the control arm non-surgical conventional medical treatment only will be used.

#### Treatment

Tonsillectomy and adeno-tonsillectomy with adenoid curettage and tonsillectomy by dissection or bipolar diathermy. Most (80%) UK surgeons use the conventional dissection method[4] and the remainder use bipolar diathermy. Both methods will be allowed in the trial according to surgical preference. Surgical intervention will take place within four weeks of randomisation.

#### Control

Non-surgical conventional medical treatment only will be used. There will be no active intervention protocol since no single prescribing strategy would be able to cover all patients[34]. The referring GP will be free to treat as in their current practice. The use of usual treatment rather than an active intervention protocol is considered important for the implementation of study findings since surgical enthusiasts may argue against the findings were the control group to be atypically and over rigorously treated.

### Outcome measurement

The primary clinical outcome is the reported number of episodes of sore throat in the two years after randomisation. Secondary clinical outcomes include reported number of episodes of sore throat, otitis media and upper respiratory tract infection which invoke a GP consultation; reported number of symptom-free days; reported severity of sore throats and surgical and anaesthetic morbidity. In addition to the measurement of these clinical outcomes, the impact of the treatment on costs and quality of life will be assessed. There will also be an economic evaluation.

### Setting

Inpatient facilities and outpatient clinics of 5 hospitals in the North of England and Scotland: Freeman Hospital, Newcastle upon Tyne; Alder Hey Children's Hospital, Liverpool; Booth Hall Children's Hospital, Manchester; Yorkhill Royal Hospital for Sick Children, Glasgow; and Bradford Royal Infirmary and general practices with which study participants are registered. Freeman Hospital, Newcastle is a large teaching hospital with a mixed adult and paediatric ENT unit. The Unit has a wide urban and rural catchment area including Newcastle and Gateshead, Northumberland and north west Durham. Alder Hey Hospital, Liverpool and Booth Hall Hospital, Manchester house two of the largest paediatric ENT units in the UK covering catchment areas in and around Liverpool and Manchester. Yorkhill is a busy university hospital with the largest children's ENT unit in Scotland and Bradford Royal Infirmary is one of the major hospitals within West Yorkshire. It has recently obtained teaching hospital status with the opening of its medical school. The ENT unit acts as a hub and supports clinics in Airedale and Dewsbury. The unit supports the majority of adult and paediatric care.

### Target population

The trial population will be children aged 4–15 on their last birthday with recurrent sore throat referred by primary care to 5 otolaryngology departments in Newcastle, Liverpool, Manchester, Glasgow and Bradford. In 1999 a total of 2683 tonsillectomy/adeno-tonsillectomy procedures were done for children in these centres: Liverpool (750), Manchester (440), Newcastle (545), Glasgow (498) and Bradford (450) of which some two-thirds will be referrals for recurrent sore throat.

#### Inclusion criteria

The study will use entry criteria drawn from the Northern regional guidelines[5]. Children (or carers) reporting experience of 4 or more episodes of sore throat within each of 2 years or 6 or more episodes of sore throat within 1 year will be eligible. We have considered pre-randomisation prospective data recording to operationalise stricter inclusion criteria for severity, but have rejected these as our aim is to operationalise current UK clinical practice.

#### Exclusion criteria

Children will be excluded if they require hospitalisation due to quinsy; have obstructive symptoms suggestive of clinically significant sleep apnoea syndrome, have rare medical conditions such as glomerulonephritis or Henoch Schonlein purpura; have previously had a tonsillectomy; have suspected velopharyngeal insufficiency, have co-morbidity that means they are unable to undergo the operation within the next 6 months, have a bleeding disorder, or have congenital/valvular heart disease.

### Number of subjects required

We estimate a completed sample size at follow up of 284 children. Allowing for an attrition rate of around 30% we will need to recruit a total of 406 children to the trial to achieve the estimated sample of 284 (who will complete the trial). Within the original three study hospitals some 1700 tonsillectomies/adeno-tonsillectomies are currently performed annually. Only two thirds of these will have recurrent sore throats. In any trial where the intervention is widely used in current practice there are likely to be large numbers of eligible participants who opt for the intervention treatment and decline participation in the trial. We estimate that this could be up to one half of all eligible referrals from primary care. The maximum available for randomisation is therefore estimated as 566 per annum. Loss of eligible subjects in the trial is expected due to holiday periods and 'winter pressures'. On the experience of loss in other trials (50%) a conservative estimate would be 283 per annum. If we assume a conservative rate of attrition of 30% over two years we would expect 198 completing trial participants to be recruited in a 12 month period. Given seasonal effects a full 2 years would be necessary to recruit the estimated sample size. The cohort sample will be identified from participants who indicated a preference not to be randomised within the trial and who agreed to data collection. An appropriate sampling fraction will be used once non-participation in the trial can be estimated.

### Subject recruitment

Recruitment to the study will take place in secondary care. All GP referrals to study centres of children with recurrent sore throat will be considered by participating surgeons. Arrangements are in place in each centre for eligible children to be referred to the clinical applicants. GPs will be informed of this reorganisation. This will facilitate efficient use of outpatients clinics at which trial participants would be recruited. Trained Research Nurses will introduce the trial to patients who will be shown a video regarding the main aspects of the trial. Patients will also receive information sheets. Research Nurses will discuss the trial with patients in light of the information provided in the video and information sheets. Patients will then be able to have an informed discussion with the participating consultant. Research Nurses will obtain written consent from patients willing to participate in the trial. Information sheets and consent forms are provided for all parents involved in the trial however these have been amended accordingly in order to provide separate information sheets and consent form which are suitable for children and teenagers. All information sheets, consent forms and the video transcript have been translated into Bengali, Punjabi, Gujarati, and Urdu. There are also separate information sheets and consent forms for the cohort group.

### Randomisation

Independent world wide web based computer randomisation will allocate participants to treatment arms. Randomisation will take place once informed consent to the study has been completed and baseline data collected. The sample will be stratified by age of child at last birthday. Blocked randomisation will be used to ensure that within each centre, within each of the three age groups (4–7, 8–11, 12–15) children will be allocated in equal numbers to each arm of the trial. Where trial sites are unable to access the world wide web they will telephone the coordinating centre (University of Newcastle) in order for web based randomisation to be completed on their behalf. Sampling for the cohort study will similarly be stratified by age.

### Blinding

Health technology assessment is essentially a pragmatic activity conducted in normal clinical practice, rather than an exploratory activity conducted in highly controlled settings. It follows that blinding doctors and patients to treatment is not desirable since it distorts normal clinical practice. Nor is it practicable. In contrast, blinding assessors is important because it minimises subjective bias towards a given treatment. All research staff conducting interviews or processing postal questionnaires and diaries will be blind to treatment modalities of all participants. This will be facilitated by separating the responsibility for recruitment and randomisation from outcome assessment. Furthermore, participants will be encouraged to respond to questions without describing their treatment regime. In this way, we will minimise subjective bias towards a given treatment.

### Data collection and follow up

All participants will be followed up for 24 months from the date of initial randomisation. To minimise recall bias, data on sore throats will be gathered by a simple, structured daily health diary completed and returned by participants on a monthly basis for 24 months. Experience of similar studies suggests that with appropriate telephone reminders 90% of diaries will be returned completed. In addition simple outcome questionnaires, using two postal reminders and a telephone reminder, will be sent to trial and cohort study participants. Overall we anticipate an 80% response rate. Postal surveys will be done at 3, 12 and 24 months after randomisation. A baseline questionnaire will be completed by all participants upon recruitment to the trial. The greater frequency of data collection in the first 12 months is necessary in order to capture data on expected changes in direct and social costs to participants in the first 12 months. Experience also suggests that data on consultation rates and prescribed medication can be gathered most accurately and reliably from medical records. Manual abstraction will be performed by trained research nurses at the end of follow up for all participants.

Adverse events will be recorded by self completion daily diaries (parent or child) which will be collected four weekly and GP records which will be examined at the end of the 24 months follow up period. Expected adverse events include infection, haemorrhage and pain following tonsillectomy with possible hospital readmission as well as sore throat, nausea, fever and dysphagia. All adverse events will be managed as per normal care, since the intervention process of this study does not deviate from normal care.

### Data handling and record keeping

Only anonymised non-identifiable data will be recorded by the site's research teams from personal medical records. Health diaries and follow-up questionnaires will be anonymous and returned to the trial centre in reply-paid envelopes. For linking purposes these data sets will have unique study identifiers. Only the lead researcher, trial manager and trial administrator will have access to the key which links study identifiers to individual data sets. Personal details (participants full name and address) will be stored on a secure database at CHSR for the purpose of sending out questionnaires and diaries centrally. All data held for analysis will be held in accordance with the Data Protection Act. On completion of the study and associated dissemination the Trial Master File will be archived in the CHSR for 10 years. Trial sites will be responsible for archiving their own documentation.

### Economic evaluation

An economic evaluation will be carried out alongside the clinical trial in order to ascertain the cost-effectiveness from a societal perspective with a focus on health service and families[35]. The cohort sample will not be included in the economic evaluation except for the purpose of validation and estimating the representativeness of cost and benefit data for trial participants.

#### Measure of benefits used and study type

Cost consequences analysis (CCA), cost-effectiveness analysis (CEA) and cost utility analysis (CUA) will be conducted. In CCA, all the outcomes used in the clinical study will be adopted as measures of benefits, including the QoL dimensions. In CEA, the benefits will be measured by the number of events of recurrent sore throat and the number of symptom-free days. In CUA, different health outcomes will be combined with QoL dimensions.

#### Resources data collected within the trial and costing methods

Medical resource data will relate to the interventions under investigation, any use of health care services due to 'sore throat' episodes not averted, treatment of drug side-effects, surgery complications and long term sequelae. Services to be monitored include: outpatient visits and hospitalisations, investigations, A&E admissions, visits and telephone consultations to and from the GP and any other health care professionals, use of medications (including antibiotics, analgesics, and drugs to manage antibiotic side-effects), and any other use of health care services in both the private and public sectors. Manpower data will be collected separately for each main category of staff. Participants' out of pocket expenses such as over the counter medicines will be reported. Costing of health care resources will be undertaken in a parallel study and a mixed approach using micro-costing and gross-costing methods will be used[36]. We will cost resources using health service pay and price data. Where appropriate, these will be integrated using national published data [37-39]. Where relevant, costs will be broken down into capital, staff, consumable and overhead costs. This will aid the production of different cost scenarios. The impact of the interventions on the time 'invested' by children and carers because of illness, treatment and rehabilitation will also be assessed. Children's days of restricted activity and their level of functioning; time off school; carers' time off work; children's and carers' time involved in outpatients attendance (such as travel time, waiting time and the duration of the clinical visit) and impact on children's and carers' quality of life will be monitored. For carers' in paid/unpaid work, time will be valued in monetary terms. Costing will be undertaken using the human capital approach and the friction cost method[40]. Those resources for which we find a statistically significant difference between the groups will be costed. Those which show no statistically significant difference but are of practical significance in their contribution to costs, will also be costed. The cost analysis will not differ across the different types of economic evaluations. However in the CUA, when carers' preferences will be assessed, particular caution will be used to avoid double counting the loss of income due to work absences[41]. Whenever applicable, a discount rate of 6% will be used, which is the rate currently used by the public sector in the UK. Costs will be expressed in UK pounds sterling. Costs will be expressed in the prices of the year in which the final analysis will be carried out and if necessary inflation method will be used to update costs data.

#### Resources/costs data collected outwith the trial

The study is not powered to detect significant differences for rare events. Given the relatively low incidence of surgical complications, long-term sequelae due to surgery and drugs side-effects, data on the related use of resources, costs to the carers and impact on children will be gathered outwith the trial, from the literature and from experts' opinions. Consensus estimates will be obtained by interviewing a panel of experts, including members of the study team and others. The source of the data will always be explicitly stated.

#### Synthesis of costs and benefits

Depending on the outcome measure, if there is no statistically significant evidence that one treatment strategy is more effective than another, a cost-minimisation framework will be used and the less expensive form of care will be recommended. If one strategy appears to be dominant (i.e. to be more effective and less costly than the alternative), the uptake will be recommended. If one form of care appears to be more effective and more expensive than the comparator, estimates of incremental cost-effectiveness (and cost-utility) ratios will be generated. A judgement will be required in a policy making context to establish whether the additional benefits should be achieved sustaining the additional costs. In any case, recommendations will be made taking into account the generalisability of the results.

#### Sensitivity analysis

To handle uncertainty not related to sampling variations and to enhance the generalisability of the results, one-way; multi-way and extreme scenario analysis will be undertaken as appropriate and confidence intervals for cost-effectiveness ratios will be estimated under different scenarios[42]. A sensitivity analysis taking into account differences in resource use which are practically significant (i.e. potentially costly) but which have not been shown to be statistically significant, will also be undertaken. The sensitivity analysis will also make explicit all the simplifying assumptions made to collect the data[43]. The application of discounting to the benefits will also be tested in the sensitivity analysis, as well as a range of discount rates. Particular attention will also be given to whether the costs data used reflect the true marginal opportunity costs of the resources used. When more than one reliable source of information is available, such data will be used as a term of comparison. The use of different costing methods for multi-centre studies will be explored. Earlier studies [28-31]. suggest that longer term outcomes such as reduction in recurrent sore throat may show only marginal benefits. An equivalence trial with a substantially larger sample size would be necessary to capture significant longer-term outcomes. To contain the cost of the trial we have not proposed a three year follow up. However, the future sequence of clinical events and economic impact will be modelled beyond 2-year follow-up. The relevant data will be derived from studies which will be available and experts' opinions.

#### Measuring participants' preferences and utilities

There is a need to value the effectiveness of interventions taking account of the risk of surgery and its long-term sequelae (e.g. sleep, eating, speech, disturbances, regressive behaviour[44]). The utility assessments will also provide insight into informed choice models[45]. Older children's and carers' values will be used to elicit preferences for trade-off between the perceived risks and benefits of surgery versus drugs treatment. Preferences will relate to temporary and chronic scenarios associated with morbidity and QoL because of symptoms and treatment complications. The scenarios will be developed selecting the health outcomes and QoL domains relevant to the problem. Interviews will be carried out with a sample of older children and carers from the cohort group, and the Standard Gamble method[46] will be used to derive utilities.

### Statistical considerations

#### Sample size calculation

In this trial we anticipate a fairly large difference in the primary clinical outcome (the reported number of episodes of sore throat in the two years after randomisation) with an effect size of around 1.0, but a smaller difference in a number of psycho-social outcomes including health-related quality of life, with an effect size of 0.33. No standard sample size formula is available for economic evaluations, and a number of methods have been proposed [47-49]. The information which is currently available limit the use of such methods in practical applications. Published data[17] suggest that tonsillectomy may lead to a reduction of approximately 1.5 days per year in missed schooling. Given a reported standard deviation of 4.5, to detect this difference with 80% power we would need approximately 142 children in each arm of the trial assuming a significance level of 5%. A sample size of 142 children in the cohort group will allow us to detect similar differences between the cohort group and propositi. The sample will be stratified by age (4–7, 8–11, 12–15). With a total of 284 children, we will have approximately 47 randomised to each treatment arm in each strata. Given that the standard deviation of the number of sore throats per year is 2.0).)18, we will be able to estimate the difference between treatments in each strata with a standard error of 0.41. (Equivalently we would have 90% power to detect a difference of 1.35 episodes of sore throat per year in each strata assuming a type 1 error of 0.05). It is anticipated that the difference in outcome between the two arms of the trial will be approximately 2 episodes in the second year of follow up. A sample size of 142 children in each arm should enable us to measure this difference with sufficient precision to undertake a meaningful economic analysis.

#### Main analysis

An intention to treat analysis will be performed. In particular, children randomised to non-surgical conventional medical treatment will be retained in that group for the analysis even if they subsequently receive a tonsillectomy. The primary clinical outcome measure will be the number of episodes of sore throat. This variable will be analysed using generalised linear modelling assuming a Poisson error structure with a log link function[50]. By fitting the difference between the two experimental groups as a fixed effect, interval estimates of the effect of tonsillectomy (in each of the first two years of follow up) will be generated. These estimates will then be used in the economic analysis. The same approach will be used to analyse the other outcomes. A Poisson error structure will be assumed for data in the form or a count (such as the number of episodes of absence from school) and normal error structure adopted for continuous variables (such as the quality of life indices).

#### Secondary analysis

The aim of secondary analysis is to determine whether we can identify groups of children who benefit from surgical treatment. It is hypothesised that disease severity may be an important factor. A severity index based on history of the condition during the year before entry to the study will be derived using data recorded in GP records. The relationship between severity and the effect of tonsillectomy will then be investigated using the modelling approach described above.

#### Economic analysis

We expect skewness in the distribution of use of resources/costs[51]. In the presence of skewness, the logarithmic transformation of data is not recommended, and the application of non-parametric tests can provide misleading results (economic studies aim to base the analysis on arithmetic means and not median values) [52,53]. The non-parametric bootstrap test can be the most appropriate[53], since it does not require any assumptions about the normality of data and equality of the variance or shape of the distributions. The t-test can be safely used if the sample size is not too small[52]. Depending on the level of skewness of data obtained we will make a judgement on which of these two methods can be safely applied. The mean costs estimates and (incremental) cost-effectiveness ratios, and conventional measures of variances will be reported[42].

#### Cohort analysis

The cohort of patients who decline to be randomised will be used to assess the external validity of the main study. Baseline characteristics of the cohort will be compared with those of the study population using standard tests for the comparison of two independent samples (e.g. the t-test or Mann-Whitney test as appropriate). Outcome for the cohort will be compared with outcome for the two groups of study participants using the modelling approach described above.

### Trial steering committee

The study has a Trial Steering Committee which meets 6 monthly. The Trial Steering Committee is responsible for monitoring public interest and ensuring issues relating to research governance are met. The trial does not have a data monitoring committee since it examines routine therapies.

### Consumer involvement

Consumer involvement will be encouraged and facilitated throughout the study by the establishment of a consumer advisory panel. We will use the advisory panel to help clarify important outcomes for children and their parents (or carers) and to assist in the development of participant-oriented data-collection methods. By consumer we include here children and their parents as well as representatives of appropriate advocacy groups such as the Patients Association. Our experience of consumer panels in the development and implementation of other studies (e.g. quality of life of people with dementia and treatment for primary biliary cirrhosis of the liver) have highlighted the different types of involvement and the different ways that consumers can be involved in primary research. Parents and children will be involved in an advisory capacity rather than in a full participatory role. We will establish and convene regularly the consumer advisory panel in which the group process will use focus group methods. Throughout the project (at least annually) we will use the advisory panel to voice participants' concerns and to identify participant-oriented solutions to such concerns.

### Ethical approval

The conduct of this study will be in accordance with the ethical principles set out in the Declaration of Helsinki. The trial has approval from MREC and all the associated LRECs. The trial also holds a Clinical Trial Authorisation from the MHRA. The trial has NHS R&D and Caldicott Guardian approval from each participating site. There are no particular ethical problems with this trial. The ethical challenge is as with any surgical randomised trial where one arm is an irreversible procedure under general anaesthesia and the other limb effectively maintenance of the status quo with reverting to surgery an outstanding choice. Set against the surgical risk, however, is the essentially curative nature of the intervention – no tonsillitis can occur once the tonsils have been removed. Further, the children under consideration all have qualifying levels of sore throat and would otherwise be eligible for surgery. In other words the issue is more the withholding of tonsillectomy rather than one of random allocation to intervention. All subjects will provide written informed consent before any study procedures are carried out and a participant information sheet will be provided. As part of the consent process participants must agree to researchers & regulatory representatives having access to their medical records. Participants will also be informed that they have the right to withdraw from the study at any time.

The NHS Trust has liability for clinical negligence that harms individuals toward whom they have a duty of care. NHS Indemnity covers NHS staff and medical academic staff with honorary contracts conducting the trial.

## Discussion

As with all pragmatic randomised controlled trials it is impossible to control the external environment in which the research is taking place. Since this trial began a number of factors have arisen which could affect the outcome. Firstly there appears to be a reduction in the incidence of respiratory tract infections or at least a reduction in the number of patients presenting to primary health care with respiratory tract infections[54]. This will inevitably lead to a reduction in the number of children being referred to secondary care for recurrent throat infections. Secondly it has come to light that there are marked socio-economic differences in consultation rates in primary health care which are not reflected in operation rates for tonsillitis in secondary care[55]. Lower socio-economic groups use NHS services for tonsillitis less in relation to need than higher socio-economic groups. Again this has implications for the rate of referral to secondary care. The results from the recent National Prospective Tonsillectomy Audit[56] may also have led to an alteration in the surgical techniques favoured by our trial consultants however surgical methods and any associated post operative complications are recorded for the trial. In addition there is anecdotal evidence that the Government's waiting list initiatives may impact the study by exporting surgery outside the NHS.

## Abbreviations

Abbreviation Definition

A&E Accident and Emergency

CCA Cost Consequences Analysis

CEA Cost Effectiveness Analysis

CHSR Centre for Health Services Research

CUA Cost Utility Analysis

ENT Ear, Nose and Throat

GP General Practitioner

LREC Local Research Ethics Committee

MREC Multi-centre Research Ethics Committee

MHRA Medicines and Healthcare products Regulatory Agency

NHS National Health Service

NvCJD New variant Creutzfeldt-Jakob Disease

UK United Kingdon

USA United States of America

## Competing interests

The author(s) declare that they have no competing interests.

## Authors' contributions

JB, JW, ME, NS, RC, AZ were involved in the original conception and design of the study. AV designed the economic evaluation. CL, KB, CS, NR were involved in the management of the trial including acquisition and interpretation of data. CL drafted the manuscript. All authors were involved in revising the manuscript critically for important intellectual content and have given approval of the final manuscript.

## Pre-publication history

The pre-publication history for this paper can be accessed here:


